# Handwashing with soap after potential faecal contact: global, regional and country estimates

**DOI:** 10.1093/ije/dyy253

**Published:** 2018-12-10

**Authors:** Jennyfer Wolf, Richard Johnston, Matthew C Freeman, Pavani K Ram, Tom Slaymaker, Eric Laurenz, Annette Prüss-Ustün

**Affiliations:** 1 Department of Public Health, Environmental and Social Determinants of Health, World Health Organization, Geneva, Switzerland; 2 Department of Environmental Health, Rollins School of Public Health, Emory University, Atlanta, GA, USA; 3 School of Public Health and Health Professions, University of Buffalo, Buffalo, NY, USA; 4 Division of Data, Research and Policy, UNICEF, New York City, NY, USA; 5 Fraunhofer ISE, Fraunhofer Institute for Solar Energy Systems, Freiburg, Germany

**Keywords:** Diarrhoea, hygiene, hand disinfection, handwashing facility, global estimates

## Abstract

**Background:**

Limited data have been available on the global practice of handwashing with soap (HWWS). To better appreciate global HWWS frequency, which plays a role in disease transmission, our objectives were to: (i) quantify the presence of designated handwashing facilities; (ii) assess the association between handwashing facility presence and observed HWWS; and (iii) derive country, regional and global HWWS estimates after potential faecal contact.

**Methods:**

First, using data from national surveys, we applied multilevel linear modelling to estimate national handwashing facility presence. Second, using multilevel Poisson modelling on datasets including both handwashing facility presence and observed HWWS after potential faecal contact, we estimated HWWS prevalence conditional on handwashing facility presence by region. For high-income countries, we used meta-analysis to pool handwashing prevalence of studies identified through a systematic review. Third, from the modelled handwashing facility presence and estimated HWWS prevalence conditional on the presence of a handwashing facility, we estimated handwashing practice at country, regional and global levels.

**Results:**

First, approximately one in four persons did not have a designated handwashing facility in 2015, based on 115 data points for 77 countries. Second the prevalence ratio between HWWS when a designated facility was present compared with when it was absent was 1.99 (1.66, 2.39) *P* <0.001 for low- and middle-income countries, based on nine datasets. Third, we estimate that in 2015, 26.2% (23.1%, 29.6%) of potential faecal contacts were followed by HWWS.

**Conclusions:**

Many people lack a designated handwashing facility, but even among those with access, HWWS is poorly practised. People with access to designated handwashing facilities are about twice as likely to wash their hands with soap after potential faecal contact as people who lack a facility. Estimates are based on limited data.

**Key Messages**

- One in four persons worldwide did not have access to a handwashing facility with soap and water on premises in 2015.
- In 2015, handwashing with soap occurred in about 26% after events of potential faecal contact, globally. In regions with high access to handwashing facilities, handwashing with soap was performed by about 51%, and in regions with more limited access, by about 22% after events of potential faecal contact.
- Though additional data and analyses are needed, quantifying the presence of handwashing facilities with soap and water on premises may be used to estimate actual handwashing behaviour.
- Important gaps exist for country-representative data on presence of designated handwashing facilities and on handwashing behaviour at household level, for all regions of the world and particularly for high-income countries.

## Introduction

Handwashing with soap (HWWS) is an important public health behaviour as it reduces exposure to faecal pathogens and other infectious agents, thereby reducing gastrointestinal^1^^,^^2^ and respiratory infections.^3^ Interventions that successfully promote HWWS are considered very cost-effective.^4^ However, HWWS was estimated to be practised in only 19% of cases after potential faecal contact, globally.^1^

The proportion of people using a handwashing facility with soap and water on premises is a global indicator of the Sustainable Development Goals (SDG indicator 6.2.1b).^5^ Country-representative data on this indicator are collected in household surveys such as demographic and health surveys (DHS) and multiple indicator cluster surveys (MICS) and assessed through observation by enumerators.^6^^,^^7^ The presence of handwashing materials at household level was associated with observed HWWS, hand cleanliness and child health.^8–10^ However, recent randomized controlled trials of interventions which provided handwashing materials and promoted handwashing with soap, combined with improvements in drinking water and sanitation, resulted in ambiguous health impacts.^11–13^ The absence of a handwashing facility with soap and water on premises does not preclude that hands are washed, but in situations where the handwashing facility is located off-site or water and soap need to be fetched, routine handwashing after potential faecal contact, or other key times such as before preparing food or eating, is less likely to occur.^8^

Handwashing practice recorded during structured observations is considered the most reliable way to measure actual handwashing behaviours,^14^^,^^15^ though some limitations exist with regard to bias.^16^ Structured observations are considered impractical to conduct routinely in national household surveys, given cost and logistical constraints.^7^^,^^16^ This study quantifies the link between observed HWWS and the presence of a designated handwashing facility, defined as a specific place within the premises of a household which has both soap and water. Previous estimates of HWWS practice were based on research studies reporting observed handwashing prevalence after potential faecal contact.^1^ These studies were usually not nationally representative, were conducted in various settings and were therefore not comparable between countries. The updatepresented here is based on nationally representative household survey data that are harmonized across countries and that cover an increasingly large number of countries around the world.

The purpose of this analysis was to quantify the current state of global handwashing practice. To that end, we had the following three objectives: (i) to quantify presence of designated handwashing facilities at national, regional and global levels; (ii) to assess the association between presence of a designated handwashing facility and observed HWWS at crucial time points; and (iii) to derive country, regional and global estimates of HWWS practice after potential faecal contact.

## Methods

**Figure 1.**
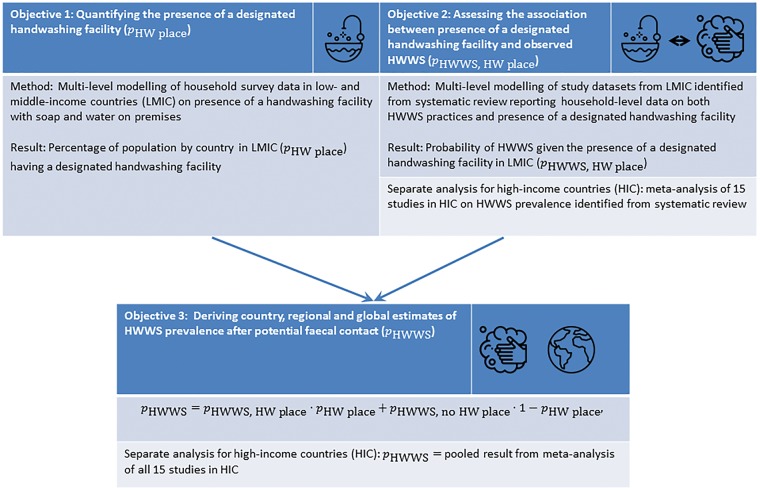
Overview of objectives, methods and results; HIC: high-income countrie; LMIC: low- and middle-income countries. Icons made by pongsakornred, Freepik, and turkkub from www.flaticon.com.

We have summarized the key methodological approaches below; but as this work uses various methods and analysis steps, we have included a figure explaining the relationship between the three objectives (Figure 1). An additional table listing the methods used for each objective in detail is provided in the Supplementary Appendix A.0.1 (available as Supplementary data at *IJE* online). We are following guidelines for accurate and transparent health estimates reporting (GATHER)^17^^,^^18^ and have included a GATHER checklist in Supplementary Appendix A.0.2. Data analysis code can be obtained from the corresponding author.

### Objective 1: quantifying the presence of a designated handwashing facility

We extracted country data on household presence of a designated handwashing facility from the WHO/UNICEF Joint Monitoring Programme for Water Supply, Sanitation and Hygiene (JMP) global database. This compiles data collected through nationally representative household surveys and censuses, and represents proportions of households that have a handwashing facility with soap and water available on premises, i.e. within the dwelling, yard or plot (defined above as and named within this manuscript as a ‘designated handwashing facility’).^19^

We modelled a continuous time series of estimates for 194 countries using a two-level (data points clustered within countries) linear multilevel model (MLM) with a random intercept by country. This approach allowed us to estimate values for countries with and without data points. The dependent variable (survey estimate of presence of a designated handwashing facility) was logit-transformed to restrict estimates between 0 and 1 (or between 0 and 100%). Covariates (fixed effects) included in the model were a continuous time variable (year, centred by its median), a factor variable indicating regional grouping (the six WHO regions: AFR, AMR, EMR, EUR, SEAR and WPR^20^) and a factor variable indicating the country’s income level. Countries’ income levels were grouped as low-, lower-middle-, upper-middle- and high-income economies for the year 2015,^21^ as the main outcome of the analysis was modelled country estimates for 2015. Urban and rural areas were modelled separately. Estimates for countries with no data point (*n* = 118, Table 1) were extrapolated from the mean urban and rural values for the respective year, region and income level (i.e. the model prediction for the fixed part). Further model details are included in Supplementary Appendix A.1.2 (available as Supplementary data at *IJE* online). Supplementary Appendix A.3.4 (available as Supplementary data at *IJE* online) lists in column H whether the modelled estimate is based on own country values or whether it was extrapolated from the mean value.

**Table 1.** Number of survey data points available by country on proportions of households with an observed designated handwashing facility

Number of countries	Number of data points^a^
117	0
48	1
23	2
4	3
1	4
1	5
Total = 194	Total = 115

a A data point comprises one estimate for urban and rural areas separately.

Confidence intervals (CIs) of the presence of a designated handwashing facility by urban and rural areas and for countries with data points were derived using the Monte Carlo method with random draws (n = 10 000) of the fixed and random effects coefficient sets and the 2.5 and 97.5 percentiles of the hereby created model predictions. For countries without data points (thus without random effect coefficients to be varied), confidence intervals were approximated as 95% prediction intervals of a standard fixed effects linear model for the respective region. The prediction interval PI was applied to the model output (main estimate y) of the MLM to yield approximate lower and upper confidence limits CL$\left(=y\pm PI/2\right)$. The MLM was implemented in Stata 14.^22^ Confidence intervals for urban and rural areas at country level were derived in R^23^ using the ‘arm’-package.^24^

Country estimates and their 95% CIs were derived using country population figures for urban and rural areas from the United Nations Population Division (2017 revision).^25^ Country estimates were calculated as population-weighted means of the urban and rural modelled values; 95% CIs were derived with standard formulae. The standard error (SE) at country level was estimated with an approach using the delta method which is described in detail here^26^ for a similar context and in the Supplementary Appendix (Equation A3), available as Supplementary data at *IJE* online. The same approach was used to calculate regional and global estimates and their 95% CIs.

As a sensitivity analysis, we modelled presence of a designated handwashing facility using only the more recent survey data since 2009, as before 2009 handwashing measurements in DHS and MICS had not yet been harmonized across countries.^7^

The predictive performance of the model was assessed using cross-validation.^27^ As modelled results are presented for the year 2015, and as it was necessary to extrapolate to many countries with no survey point at all, leave-one-out cross validation was used for the number *n* of countries with a survey point for the year 2015 (*country_1_,…, country_n_*). The model was fitted *n* times, each time setting all survey points for *country_i_* as missing (*country_i_* formed the ‘test set’ in round *n*). Subsequently, *country_i_* model prediction was compared with *country_i_* actual survey value for the year 2015 and the root mean squared error across *n* rounds was calculated.

### Objective 2: assessing the association between presence of a designated handwashing facility and observed HWWS

#### Systematic review of observed HWWS prevalence after potential faecal contact

We updated the results of our previous systematic review^1^ to identify any study that reported proportion of observed HWWS after potential faecal contact, published from August 2013 to February 2016, using PubMed, Embase and ISI Web of Knowledge. No restrictions were placed on language or study type. The search strategy was identical to that in the previous review and used the following keywords: [observ*] AND [hand wash*], [handwash*], [soap]. The database search was supplemented with data identified in a previous review^28^ and with additional Google Scholar searches of author names identified during the systematic database search. In addition, subject matter experts were contacted for unpublished handwashing observations.

We included any study that reported HWWS practice assessed with structured observations in adults and children. Though direct observation has shown to change participant behaviours,^16^ this bias is lower than what has been found with self-report.^29^ Therefore, studies were sought that reported the observed prevalence of HWWS after using a toilet or after potential contact with human excreta (including children’s excreta). There are several key events for HWWS, including food preparation, eating or feeding a child, but we focused on HWWS after potential contact with faecal pathogens such as after defecation, after using the toilet and after potential contact with child faeces. Contrary to our previous systematic review, we excluded handwashing observation studies in day care centres and schools (pre-, primary, middle and high school) and refugee camps, as such settings might not be representative for handwashing at household level.

Studies were selected for inclusion using a two-step review process. Titles and abstracts of all studies identified in the search were screened for relevance. The full text of each of the relevant articles was then reviewed, and studies were excluded if they did not provide observational data on the prevalence of observed HWWS. Data were extracted from each study using a standard protocol and included information on study setting (country), observation location (home or public setting), time frame of survey, population subgroup, sample size, a description of how handwashing prevalence was measured and specific prevalence estimates for any of the handwashing occasions, such as after toilet use or after cleaning up after a child. Characteristic of included studies and their references are listed in Supplementary Appendix A.2.1 (available as Supplementary data at *IJE* online).

#### Multilevel analysis of the association between presence of a designated handwashing facility and observed HWWS

##### Inclusion criteria and study selection

For analysing the association between presence of a designated handwashing facility and HWWS, studies were assembled from the systematic review that reported both observed presence of a designated place for handwashing and HWWS at critical times (i.e. after faecal exposure and before food contact), assessed through structured observations. Additional study datasets were provided from subject matter experts. Studies needed to fulfil the following further criteria to be included in the analysis: (i) the study needed to be conducted at household-level; (ii) in case of post-intervention data, the control group needed to be identifiable; and (iii) the study needed to have been conducted within the past 20 years (1998–2017).

In case of intervention data, we included data from both intervention and control groups at study baseline if available (e.g. Nepal, Zimbabwe^30^^,^^31^). However, in most studies data on the observed designated handwashing facility and structured observations of HWWS were available post-intervention only. From these studies, we used post-intervention data and we used data from control households only. For the Zimbabwe study,^31^ data on observed designated handwashing facilities and HWWS were available at study baseline and post-intervention. Here we added post-intervention data from the control group in case these households had not been included at baseline.

##### Exposure definition

The exposure of interest was defined as an observed designated handwashing facility (defined above as a designated place within the premises of a household which has both soap and water) that could include a specific handwashing hardware, such as a tippy tap or washbasin, or be any set place that householders consider their designated handwashing place. Soap in this context includes any kind of soap or detergent but excludes mud, sand or ash. In most studies, householders were asked to show the place where household members usually washed hands and the presence of soap and water was observed. In two datasets, information on the designated handwashing facility was given for the primary and secondary (or tertiary) handwashing facility (Nepal, Tanzania). In these datasets, the main exposure was present (coded 1) when soap and water were present at any of the observed handwashing facilities.

##### Outcome definition

The primary outcome was observed HWWS after potential faecal contact. Both hands needed to be washed with soap. Potential faecal contact in this analysis includes visiting the toilet, defaecation and cleaning a child’s bottom or changing its nappies, but does not include potential contact with animal faeces. Any handwashing occasion by any household member reported in the primary studies and related to the respective exposure was included in the analysis. Though the literature search had focused on observed HWWS after potential faecal contact, we also analysed the available data to investigate the association between presence of a designated handwashing facility and observed HWWS before food contact. Food contact in this analysis includes preparing, cooking or serving food, eating, and feeding or breastfeeding a child. We excluded handwashing observations when either the designated handwashing facility or HWWS could not be observed.

##### Statistical analysis

To estimate adjusted prevalences and prevalence ratios from binary outcome data, we used Poisson regression with robust standard errors.^32^^,^^33^ We used a three-level model (handwashing observations clustered within households, households clustered within countries (or studies) to estimate HWWS prevalence after potential faecal contact, and to account for the clustering of observations within households and countries (or studies). HWWS was included as a binary variable and coded ‘1’ if HWWS occurred after potential faecal contact and coded ‘0’ if the respective contact occurred but hands were not washed or not washed with soap or only one hand was washed with soap. Presence or absence of a designated handwashing facility was included as a binary variable. Absence includes presence of a handwashing facility on premises that are not equipped with soap and water, as well as no place for handwashing on premises. As further fixed effect covariates, we included binary variables for the respective WHO region^20^ (Supplementary Appendix 1.1, available as Supplementary data at *IJE* online). As determinants for washing own hands might be different from washing children’s hands, we analysed data restricted to handwashing occasions among adults. More details on the model are included in Supplementary Appendix A.2.3 (available as Supplementary data at *IJE* online).

We used the ‘margins’ command in Stata^34^ to predict adjusted HWWS prevalence after potential faecal contact at regional level for households with and without a designated handwashing facility, for each region represented by included studies (AFR, AMR, SEAR, and WPR).^34^ We predicted the average adjusted HWWS prevalence after potential faecal contact for households having or not having a designated handwashing facility for the remaining regions (low- and middle-income countries only) not represented by included studies (EMR and EUR).^34^ To account for the uncertainty of estimates for the latter, 95% prediction intervals were approximated based on the standard deviation of the available country-level adjusted prevalences. Analyses were performed in Stata 14.^22^

#### High-income countries: meta-analysis of observed HWWS prevalence after potential faecal contact

As the selected studies for the analysis of the association between presence of a designated handwashing facility and observed HWWS were conducted exclusively in low- and middle-income countries, we conducted random effects meta-analysis on observed HWWS prevalence after potential faecal contact in high-income country studies identified in the systematic review, to approximate the proportion of HWWS after potential faecal contact in those countries.

### Objective 3: deriving country, regional and global estimates of HWWS prevalence after potential faecal contact

To estimate HWWS prevalence $p_{\text{HWWS}}$ after potential faecal contact at country level for low- and middle-income countries in 2015, we applied the following formula**:**

(1) $$p_{\text{HWWS}}=p_{\text{HWWS},\;\text{HW}\;\text{place}}\cdot p_{\text{HW}\;\text{place}}+p_{\text{HWWS},\;\text{no}\;\text{HW}\;\text{place}}\cdot 1-p_{\text{HW}\;\text{place}},$$

where $p_{\text{HWWS},\;\text{HW}\;\text{place}}$ is the estimated HWWS prevalence after potential faecal contact in the presence of an observed designated handwashing facility (estimated at regional level, Table 4), the proportion $p_{\text{HW}\;\text{place}}$ is the modelled estimate of presence of a designated handwashing facility at country level and $p_{\text{HWWS},\;\text{no}\;\text{HW}\;\text{place}}$ is the estimated HWWS prevalence after potential faecal contact without having a designated handwashing facility (Tables 2 and 4). The estimates for $p_{\text{HWWS},\;\text{HW}\;\text{place}}$ and $p_{\text{HWWS},\;\text{no}\;\text{HW}\;\text{place}}$ at country level are extrapolated from modelled results at regional level (see objective 2). This projection from regional to country level results in an increase of the confidence intervals, just as the inverse operation—the aggregation from country to regional level—would lead to a decrease, as the uncertainties at country level would partially offset each other (Supplementary Appendix Equations A3 and A.3.1, available as Supplementary data at *IJE* online). For estimating HWWS prevalence after potential faecal contact for high-income countries, the pooled HWWS prevalence from the meta-analysis (objective 2) was taken. To estimate the proportion of potential faecal events that were followed by HWWS at regional and global level for low-, middle- and high-income countries, we calculated population-weighted means of the country estimates. As we do not know the total number of potential faecal contacts by country and region, we assume an equal average number of faecal contacts by person across countries and regions, and use total population by country and region to calculate the regional and global estimates. The calculation of the 95% confidence intervals for these estimates at country, regional and global levels is described in Supplementary Appendix A.3.2, A.3.3 (available as Supplementary data at *IJE* online).

**Table 2.** Percentage of population having access to a designated handwashing facility in 2015, by area

Area	Percentage of population (95% CI) with access to a designated handwashing facility in 2015
AFR LMI	17.7 (14.8, 21.0)
AMR LMI	83.3 (72.2, 90.5)
EMR LMI	68.3 (59.2, 76.1)
EUR LMI	95.7 (91.8, 97.8)
SEAR LMI	69.4 (39.5, 88.7)
WPR LMI	89.6 (67.7, 97.3)
Urban	84.1 (75.6, 90.1)
Rural	61.2 (40.7, 78.3)
Low- and middle-income countries	69.5 (56.8, 79.8)
High-income countries	95.0 (89.8, 97.7)
World	73.5 (63.2, 81.8)

AFR, WHO African Region; AMR, WHO Region of the Americas; EMR, WHO Eastern Mediterranean Region; EUR, WHO European Region; SEAR, WHO South-East Asia Region; WPR, WHO Western Pacific Region; LMI, low- and middle-income.

As a sensitivity analysis, we re-calculated regional and global handwashing prevalence, applying the approach for low- and middle-income countries to high-income countries. We did so by using formula 1 for high-income countries, where $p_{\text{HW}\;\text{place}}$ is the modelled estimate of presence of a designated handwashing facility for high-income countries, $p_{\text{HWWS},\;\text{HW}\;\text{place}}\;$is the pooled estimate of handwashing prevalence from meta-analysis from all handwashing observation studies in high-income countries and $p_{\text{HWWS},\;\text{no}\;\text{HW}\;\text{place}}$was approximated with the mean handwashing prevalence without a designated handwashing facility from the analysis of the nine low- and middle-income country datasets (Table 4).

**Table 3.** Characteristics of included datasets for estimating the association between presence of a designated handwashing facility and observed HWWS

Country (WHO region)	Year	Setting	Interventions	Baseline/endline	Household member role (for SO)	Sample size	HWWS after faecal contact	References
Ethiopia (AFR)	2012/ 2013	Rural	Four hygiene arms against control	HW place endline only (self-reported in baseline), so base- & endline	Mixed	59 households, 78 faecal contacts(controls)	7.7%	^36^
Senegal (AFR)	2011	Urban/rural	Hygiene arm against control	SO endline only, HW place base- & endline	Mixed (primary caregiver, other adults, children)	88 households, 231 faecal contacts (controls)	14.3%	^37^
Tanzania (AFR)	2012	Rural	Three intervention arms (hygiene, sanitation & hygiene + sanitation) against control	Only endline data	Mixed	185 households, 239 faecal contacts (controls)	12.1%	^38^
Zimbabwe (AFR), two datasets included	urban: 2014 (baseline)/2015 (endline); rural: 2016 (baseline)/2017 (endline)	Urban & rural dataset	Hygiene arm against control (SO) (with pre-intervention data) (intervention and control group included)	SO and HW place base- & endline	Mixed (primary caregiver, other adults, children)	211 rural + 388 urban households, 322 rural + 432 urban faecal contacts(all groups at baseline, controls at endline)	4.6% total (5.3% rural, 4.2% urban)	^31^
Peru (AMR)	2011	Rural	Two intervention arms against control	SO endline only, HW place base- & endline	Mixed (primary caregiver, other adults, children)	282 households, 421 faecal contacts	33.3%	^39^
Bangladesh (SEAR)	2013	Urban	Two intervention arms (vaccination & vaccination + hygiene) against control	SO endline only, HW base- & endline	Primary caregiver	112 households, 39 faecal contacts	18%	^40^
Nepal (SEAR)	2012	Rural	Baseline data (intervention and control group included)	Only baseline data	Mixed (primary caregiver, other adults, children)	1008 households, 1690 faecal contacts	23.7%	^30^
Viet Nam (WPR)	2010/ 2011	Rural	Two hygiene arms against control	SO endline only, HW place base- & endline	Primary caregiver and children up to 5 years	200 households, 501 faecal contacts (controls)	16.8%	^41^

AFR, WHO African Region; AMR, WHO Region of the Americas; EMR, WHO Eastern Mediterranean Region; EUR, WHO European Region; SEAR, WHO South-East Asia Region; WPR, WHO Western Pacific Region; HW, handwashing; SO, structured observations; HWWS, handwashing with soap.

**Table 4.** Predicted HWWS prevalence by presence of a designated handwashing facility and by region

	Predicted HWWS (95% CI) after potential faecal contact (proportion)	
Area	In households without designated handwashing facility	In households with designated handwashing facility
AFR, LMI	0.071 (0.033, 0.110)	0.142 (0.056, 0.227)
AMR, LMI	0.198 (0.165, 0.231)	0.394 (0.362, 0.426)
EMR, LMI	0.128 (0.040, 0.337)	0.254 (0.078, 0.578)
EUR, LMI	0.128 (0.040, 0.337)	0.254 (0.078, 0.578)
SEAR, LMI	0.163 (0.144, 0.182)	0.325 (0.277, 0.373)
WPR, LMI	0.090 (0.074, 0.107)	0.180 (0.167, 0.193)
High-income countries	0.506 (0.426, 0.585)^a^	

Values for LMI regions are predicted adjusted prevalences at representative (regional) values (AFR, SEAR and WPR) and average (pooled across regions) adjusted prevalence (AMR, EMR and EUR) calculated from the multilevel logistic model.

**Figure 2.**
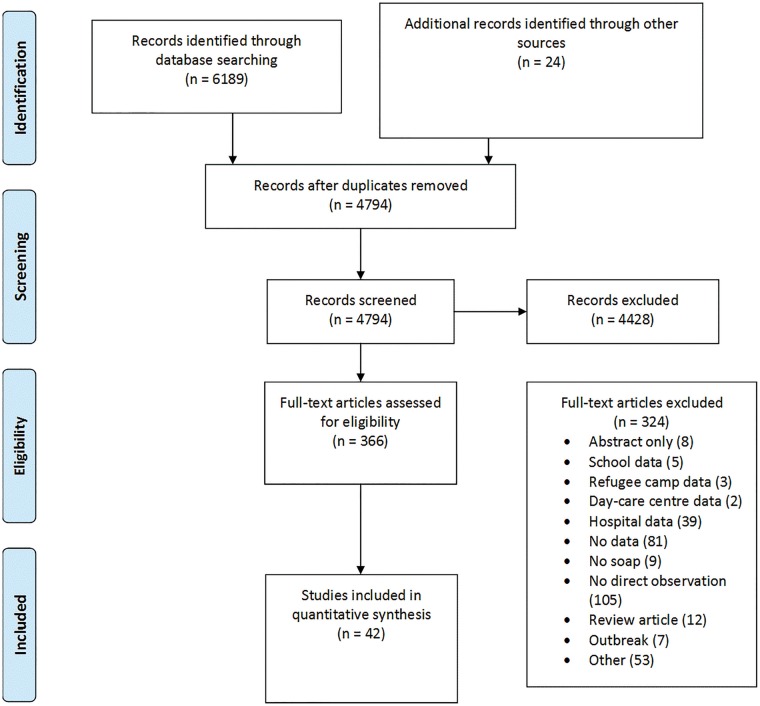
PRISMA flow chart^35^ of selection process of handwashing observation studies.

HWWS, handwashing with soap; AFR, WHO African Region; AMR, WHO Region of the Americas; EMR, WHO Eastern Mediterranean Region; EUR, WHO European Region; SEAR, WHO South-East Asia Region; WPR, WHO Western Pacific Region; LMI, low- and middle-income.

a Value taken from meta-analysis on studies in high-income countries (objective 2, Figure 2).

## Results

### Objective 1: quantifying the presence of a designated handwashing facility

#### Data availability

The dataset consisted of 115 data points for urban and rural areas each, over the time period 2000 to 2016. A total of 77 countries had at least one data point available, including 76 low- and middle-income countries [of those, 38 countries in the WHO African Region (AFR), 14 countries in the WHO Region of the Americas (AMR), eight in the WHO Eastern Mediterranean Region (EMR), eight in the WHO European Region (EUR), five in the WHO South-East Asia Region (SEAR) and three in the WHO Western Pacific Region (WPR)] and one high-income country (Barbados).^20^ More information on the regional grouping is listed in Supplementary Appendix A.1.1, available as Supplementary data at *IJE* online). Only 29 countries had more than one data point available, making it difficult to determine trends (Table 1).

#### Modelled results of handwashing facility presence

Table 2 lists percentage of population having access to a designated handwashing facility by area. We estimate that in 2015, 73.5% of the world population had access to a designated handwashing facility. In high-income countries, 95% of the population have access to a designated handwashing facility compared with 69.5% in low- and middle-income countries. A similar difference is estimated between urban and rural areas, with 84.1% in urban compared with 61.2% in rural areas.

The root mean squared error (RMSE) from cross-validation was 11.1% for urban and 7.3% for rural areas, indicating that on average the model estimate for the year 2015 for a country without any survey point is respectively 11.1 and 7.3 percentage points away from the ‘true’ value (which is approximated here with the survey point for 2015 for the respective country).

When modelling the presence of a designated handwashing facility based on uniformly collected data in MICS/ DHS from 2009 onwards (removing 13 data points for both urban and rural areas which originated from before 2009), the estimated percentage of population with access to a designated handwashing facility changed only slightly (AFR LMI: 17.1; AMR LMI: 83.0; EMR LMI: 66.4; EUR LMI: 95.9; SEAR LMI: 68.3; WPR LMI: 89.8; urban: 84.1; rural: 60.1; low- and middle-income countries: 68.9; high-income countries: 94.9; world: 73.0).

### Objective 2: assessing the association between presence of a designated handwashing facility and observed HWWS

#### Data availability

The systematic review identified a total of 42 studies of which 15 were conducted in high-income countries (Figure 2). Nine datasets (five studies were identified through the systematic database search and three that had not been published at the time of the analysis were provided from subject matter experts) from eight low- and middle-income countries, in four out of six WHO regions, reported both presence of a designated handwashing facility and structured observations of handwashing after potential faecal contact, and thus were included in the analysis of objective 2.

#### Multilevel analysis of the association between presence of a designated handwashing facility and observed HWWS

The eight studies (providing nine datasets), reporting both presence of a designated handwashing facility and structured observations of handwashing after potential faecal contact, were combined to estimate the association between presence of a designated handwashing facility and HWWS after potential faecal contact. Data from Zimbabwe urban and rural sites were different parts of one study from the same research group. Table 3 lists characteristics of included studies. Further suitable studies that were identified from the systematic search but not included in the analysis are listed, with reasons for exclusion, in Supplementary Appendix A.2.4 (available as Supplementary data at *IJE* online).

The pooled dataset includes 3953 observed potential handwashing occasions after potential faecal contact. Of those, hands were washed with soap in 18.6% of potential handwashing occasions. In the presence of a designated handwashing facility, hands were washed with soap in 25% of potential handwashing occasions compared with 12% in the absence of a designated facility. The 28 observations with missing values in the variable indicating the presence of a handwashing place with soap and water (0.7% of all observations) were excluded from the analysis (i.e. listwise deletion).

From the three-level Poisson model there was very strong evidence (*P* <0.001) for an association between presence of a designated handwashing facility and observed HWWS after potential faecal contact in structured observations. After taking account of the clustering of observations in households and countries (or studies), and after adjusting for study region, the prevalence of HWWS where a designated handwashing facility was present was 1.99 (95% CI 1.66, 2.39) times higher than in the absence of a designated facility, Note that in the absence of a designated handwashing facility, householders could still fetch water and soap to wash their hands, use the neighbour’s facility etc..

There was also very strong evidence (*P* <0.001) for an association between having a designated handwashing facility and observed HWWS before food contact (there were a total of 12 052 potential handwashing occasions before food contact, of which hands were washed in 5%). After taking account of the clustering of observations in households and countries (or studies) and after adjusting for study region, the prevalence of HWWS in presence of a designated handwashing facility was present was 2.57 (95% CI 2.26, 2.92) times higher than in the absence of a designated facility.

After restricting to adult handwashing, there remained 2578 observed potential handwashing occasions after potential faecal contact and 7554 potential handwashing occasions before food contact. Of those, hands were washed with soap in 19.1% of potential handwashing occasions after potential faecal contact and in 4% of potential handwashing occasions before food contact. The association between presence of a designated handwashing facility and adult HWWS after potential faecal or before food contact remained very strong (*P*  <0.001) and of similar size compared with the analysis including data on children’s handwashing (prevalence ratios for HWWS after potential faecal contact 2.02 (95% CI 1.77, 2.32) and before food contact 2.42 (1.96, 3.00)).

#### High-income countries: meta-analysis of HWWS prevalence after potential faecal contact

**Figure 3.**
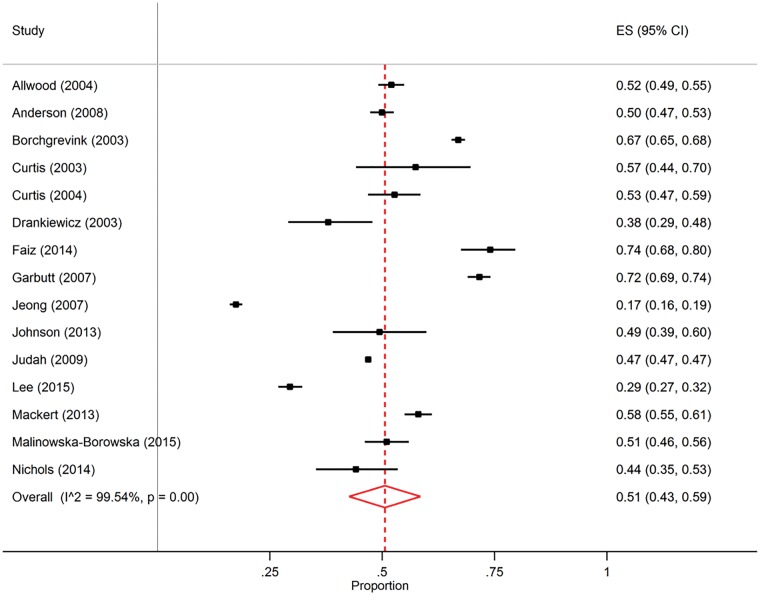
Forest plot of handwashing observation studies in high-income countries.

Supplementary Appendix A.2.1 (available as Supplementary data at *IJE* online) includes citations, number of observed HWWS events after potential faecal exposure, number of observed faecal contact events and characteristics for all 15 studies in high-income countries included in random effect meta-analysis. The pooled HWWS proportion after potential faecal contact was 51% (95% CI: 43%, 59%) (independent of presence of a designated handwashing facility, Figure 3). The I^2^ of 99.5% indicates high heterogeneity of HWWS between studies.^42^ Subgroup meta-analysis by study region did not lead to a relevant reduction of I^2^ (>95% within subgroups), which resulted in the choice of the pooled estimate over all 15 studies. We additionally examined the 15 studies for potential outliers.^43^ Jeong 2007^44^ was identified as outlier, and the removal of this study changed the pooled estimate from 51% to 53%. A forest plot of the meta-analysis of all studies identified in the systematic review, separately by high- versus low- and middle-income countries, is placed in Supplementary Appendix A.2.2 (available as Supplementary data at *IJE* online).

#### HWWS prevalence results by presence of a handwashing facility and by region

Adjusted HWWS prevalences after potential faecal contact by presence of a designated handwashing facility and by region are given in Table 4. Predicted HWWS prevalences by presence/absence of a designated handwashing facility for low- and middle-income regions are derived from the analysis described in objective 2. Proportions of HWWS after potential faecal contact for high-income regions are taken from the meta-analysis of all identified studies in high-income countries described in the same objective. Supplementary Appendix A.3.4 (available as Supplementary data at *IJE* online) lists under column I how HWWS estimates were generated by country.

### Objective 3: deriving country, regional and global estimates of HWWS prevalence after potential faecal contact

HWWS prevalences after potential faecal contact, by country for low- and middle-income countries, are computed on the basis of the estimates of presence of a designated handwashing facility at country- evel (objective 1) and the adjusted regional predictions of HWWS after potential faecal contact by presence/absence of a designated facility (objective 2, Table 4) using formula 1. For high-income countries, handwashing prevalence was estimated using the result from meta-analysis (objective 2, Figure 2).

Using this approach, we estimate that, worldwide, 26.2% of potential faecal contacts were followed by HWWS in 2015 (Table 5). Country estimates for HWWS after potential faecal contact are provided in Supplementary Appendix A.3.4 (available as Supplementary data at *IJE* online).

**Table 5.** Percentage of faecal contacts that are followed by HWWS (in 2015), by area

Area	Percentage (95% CI) of faecal contacts followed by HWWS
AFR, LMI	8.4 (5.0, 13.6)
AMR, LMI	36.1 (33.0, 39.4)
EMR, LMI	21.4 (8.2, 45.2)
EUR, LMI	24.9 (7.8, 56.3)
SEAR, LMI	27.6 (22.5, 33.3)
WPR, LMI	17.1 (15.5, 18.7)
Low- and middle-income countries	21.7 (18.7, 25.1)
High-income countries	50.6 (42.6, 58.5)
World	26.2 (23.1, 29.6)

The estimate for low- and middle-income countries is the combined estimate from the six regions above.

HWWS, handwashing with soap; AFR, WHO African Region; AMR, WHO Region of the Americas; EMR, WHO Eastern Mediterranean Region; EUR, WHO European Region; SEAR, WHO South-East Asia Region; WPR, WHO Western Pacific Region; LMI, low- and middle-income.

When calculating the regional (high-income countries) and global proportions of faecal contacts followed by HWWS using formula 1 as described above (sensitivity analysis described in Methods) also for high-income countries, estimates changed only slightly (48.7% instead of 50.6% for high-income countries and 25.9% instead of 26.2% for the world).

## Discussion

This analysis shows that 27% of the world population—nearly two billion people—lack a designated handwashing facility (objective 1). People who have access to a designated handwashing facility, defined here as a specific place within the premises of a household which has both soap and water, are about twice as likely to wash hands with soap after potential faecal contact and 2.6 times as likely to wash hands with soap before food contact, compared with people without a designated facility (objective 2). Only 26% of potential faecal contacts (i.e. after visiting the toilet, after defaecation, after cleaning a child’s bottom or changing its nappies) were followed by HWWS (objective 3).

We provide country, regional and global HWWS estimates after potential faecal contact. These estimates are based on modelled estimates of survey data on the presence of designated handwashing facilities. The modelled estimates were converted to estimated HWWS practice using predicted handwashing prevalence in the presence or absence of a designated handwashing facility.

Compared with our previous estimate of 19% HWWS after potential faecal contact,^1^ the estimates from the current analysis presented here are slightly higher. Whereas it is possible that a real global trend exists due to efforts of improving HWWS after potential faecal contact globally,^45^ it is more likely that this new estimate represents our revised and improved modelling approach. We use a revised and extended methodology compared with our assessment in 2012 which had relied solely on a systematic review of observed HWWS. We now use country-representative data on presence of essential handwashing materials at a designated handwashing facility, converting them into handwashing practice based on an analysis of the association between presence of a designated handwashing facility and handwashing behaviour. Many more countries are now covered by national data compared with the previous approach (76 versus 28 low- and middle-income countries).

### Strengths and limitations

#### Objective 1

The estimates for presence of a designated handwashing facility are based on 115 data points. For 118 of 194 countries there was no data point, and for 48 countries there was only one data point. Trends over time were estimated based on the overall trend from available data points. For countries with no data, the mean for the respective region and income group was used. As such, the reliability of estimates for different regions and countries varies. Results from cross-validation showed, however, that the model predicted was sufficiently close to the actual survey data point even for countries with no data point. Our modelling approach offers several advantages, including a single model for all countries, a continuous time series and the use of information from other country data for countries with few data or no data.

#### Objective 2

Estimates for the association between presence of a designated handwashing facility and observed HWWS are derived by regions and are based on nine datasets from eight heterogeneous studies in low- and middle-income countries, covering various subgroups of populations in four out of six regions. For EMR and EUR regions, HWWS practice was assumed as the average adjusted predicted prevalence based on all included studies. As such, estimates of the association between presence of a designated handwashing facility and observed HWWS are based on limited evidence. However, the proportion of observed HWWS is consistently low across the nine datasets (Table 3). The African region is covered by four datapoints which are all lower than any other datapoint for any different region. Removing one of the datasets at a time changed the predicted mean HWWS after potential faecal contact only to a minor extent [from 0.25 predicted HWWS in households with a basic handwashing facility, including all studies to a minimum of 0.2 (excluding Nepal), and to a maximum of 0.27 (excluding Viet Nam)]. Furthermore, overall handwashing prevalence after potential faecal contact—irrespective of handwashing facility presence—across the nine datasets, taking account of clustering within studies, is 14%, which is close to the pooled estimate of 17% from all 27 observations from low- and middle-income countries. This indicates that HWWS rates across these settings are consistently low (forest plot in Supplementary Appendix A.2.2, available as Supplementary data at *IJE* online).

Of the 15 studies conducted in high-income countries, there was only one^46^ that was conducted at household level; the rest were conducted in public settings, which may limit their generalizability to the household setting. In the one household-level study, hands were washed with soap in about 80% after visiting the toilet but only in 42% after changing a dirty nappy.^46^ Studies conducted in public settings will mostly include potential handwashing occasions after visiting the toilet, whereas household-level studies will also include occasions after cleaning a child’s bottom or changing its nappies. In the household-level study, 57% of potential faecal contacts were followed by HWWS, which is however close to the pooled estimate of 51% for all high-income countries, and close to the handwashing prevalence found for a study conducted in the same country but in a public setting.^47^ The *I^2^* statistic, a measure of inconsistency across study findings,^42^ was very high, which indicates that results for individual high-income countries might vary to a larger extent from the pooled estimate, and likely reflects substantial differences among the studies in terms of study design and methods, settings and populations.

#### Objective 3

This study provides important input to the SDG indicator 3.9.2 (Mortality from water, sanitation and hygiene),^5^estimating population exposures to inadequate handwashing practices. HWWS estimates are based on a novel approach using country-representative data on presence of a designated handwashing facility, converting them into actual handwashing practice. We assumed that the average number of potential faecal contacts per person was the same across regions but might not be correct for different reasons, e.g. different number of children per caregiver. Due to data scarcity for high-income countries, the estimate of HWWS for high-income countries is not based on modelled estimates from national household surveys (as are the estimates for low- and middle-income countries), but only on smaller observational studies reporting handwashing behaviour. The sensitivity analysis using modelled estimates for high-income countries yielded, however, very similar estimates for HWWS practice.

### Further discussion

This analysis proposes an approach for estimating HWWS practice after potential faecal contact, including after toilet use, which is based on adjusted nationally representative household survey data and the probability of HWWS by presence/absence of a designated handwashing facility.

We quantify the presence and importance of designated handwashing places as a crucial first step for achieving the desired handwashing behaviour. This analysis suggests that data on the presence of designated handwashing facilities at household level could be used to assess actual handwashing behaviour when handwashing facility presence is adjusted by the association with actual observed handwashing. This could be highly valuable for aggregated HWWS estimates (i.e. at country level) because data on handwashing facility presence are reliable and efficient and—whereas structured observations can estimate handwashing behaviour directly—structured observations are also too time consuming to be integrated in national data collection.^6^^,^^14^ This analysis also suggests that using handwashing facility presence for estimating handwashing behaviour, without further adjustment, would grossly overestimate actual handwashing prevalence, as we show that in low- and middle-income countries only 25% of potential faecal contacts in households with access to a designated handwashing facility are followed by HWWS. We show that HWWS practice after potential faecal contact is low, in particular in low- and middle-income-countries but also in high-income countries. There are further substantial differences between HWWS in sub-Saharan Africa (WHO AFR region) and low- and middle-income countries from other regions. In sub-Saharan Africa, access to designated handwashing facilities is much lower than in other world regions (Table 2). Even in the presence of a designated handwashing facility, HWWS was lower in sub-Saharan Africa compared with other low- and middle-income regions (12% versus 29% of all potential faecal contacts). Given that HWWS is associated with the risk of diarrhoea and acute respiratory infections,^48^^,^^49^ it is important to take action at national and international levels to reduce this important global public health risk.

## Conclusions

HWWS is practised in around 26% of cases after potential faecal contact. To effectively promote HWWS, there is a need to increase handwashing facilities equipped with soap and water in or around the home. As HWWS remains limited even in the presence of a designated handwashing facility, efforts to promote handwashing behaviour need to be conducted simultaneously. This analysis uses nationally representative survey data and derived HWWS estimates, and is in line with individual HWWS observation studies. It suggests that the presence of a designated handwashing facility could be used to estimate actual handwashing behaviour. However, our estimates are based on limited data, i.e. 115 survey data points covering 77 countries for access to designated handwashing facilities and nine datasets from eight low- and middle-income countries, to analyse the association between presence of a designated handwashing facility and observed handwashing behaviour. There is a need for greater data availability on hand hygiene, in particular for: (i) data at household level reporting observed handwashing behaviour, including reporting of the presence of handwashing facilities for low-, middle- and especially high-income countries; and (ii) nationally representative data on presence of handwashing facilities for high-income countries and those low- and middle-income countries not yet covered by such data.

## Funding

This work was supported by the United Kingdom Department for International Development (DFID) [204700–101]. Those acknowledged have confirmed their agreement.

## Supplementary Material

dyy253_Supplementary_Data

## References

[1] Freeman MC, Stocks ME, Cumming O et al Hygiene and health: systematic review of handwashing practices worldwide and update of health effects. Trop Med Int Health 2014;19:906–16. 24889816 10.1111/tmi.12339

[2] Ejemot-Nwadiaro RI, Ehiri JE, Arikpo D, Meremikwu MM, Critchley JA. Hand washing promotion for preventing diarrhoea. Cochrane Database Syst Rev 2015;9:CD004265. 10.1002/14651858.CD004265.pub3 PMC4563982 26346329

[3] Rabie T, Curtis V. Handwashing and risk of respiratory infections: a quantitative systematic review. Trop Med Int Health 2006;11:258–67. 16553905 10.1111/j.1365-3156.2006.01568.x PMC7169664

[4] Townsend J, Greenland K, Curtis V. Costs of diarrhoea and acute respiratory infection attributable to not handwashing: the cases of India and China. Trop Med Int Health 2017;22:74–81. 28043097 10.1111/tmi.12808

[5] United Nations. Sustainable Development Goals. 2015 http://www.un.org/sustainabledevelopment/sustainable-development-goals/ (18 October 2016, date last accessed).

[6] Kumar S, Loughnan L, Luyendijk R et al Handwashing in 51 countries: analysis of proxy measures of handwashing behavior in multiple indicator cluster surveys and demographic and health surveys, 2010–13. Am J Trop Med Hyg 2017;97:447–59. 28722572 10.4269/ajtmh.16-0445 PMC5544068

[7] Loughnan LC, Ram PK, Luyendijk R. Measurement of handwashing behaviour in multiple indicator cluster surveys and demographic and health surveys, 1985–2008. Waterlines 2015;34:296–13. 10.4269/ajtmh.16-0445 PMC5544068 28722572

[8] Ram PK, Sahli M, Arnold B et al Global Scaling up Handwashing: Validity of Rapid Measures of Handwashing Behavior: An Analysis of Data From Multiple Impact Evaluations in the Global Scaling up Handwashing Project. Washington, DC: Water and Sanitation Program, World Bank Group, 2014.

[9] Halder AK, Tronchet C, Akhter S, Bhuiya A, Johnston R, Luby SP. Observed hand cleanliness and other measures of handwashing behavior in rural Bangladesh. BMC Public Health 2010;10:1. 20828412 10.1186/1471-2458-10-545 PMC2944374

[10] Luby SP, Halder AK. Associations among handwashing indicators, wealth, and symptoms of childhood respiratory illness in urban Bangladesh. Trop Med Int Health 2008;13:835–44. 18363587 10.1111/j.1365-3156.2008.02074.x

[11] Null C, Stewart CP, Pickering AJ et al Effects of water quality, sanitation, handwashing, and nutritional interventions on diarrhoea and child growth in rural Kenya: a cluster-randomised controlled trial. Lancet Glob Health 2018;6:e316–29. 29396219 10.1016/S2214-109X(18)30005-6 PMC5809717

[12] Stewart CP, Kariger P, Fernald L et al Effects of water quality, sanitation, handwashing, and nutritional interventions on child development in rural Kenya (WASH Benefits Kenya): a cluster-randomised controlled trial. Lancet Child Adolesc Health 2018;2:269–80. 29616236 10.1016/S2352-4642(18)30025-7 PMC5859215

[13] Luby SP, Rahman M, Arnold BF et al Effects of water quality, sanitation, handwashing, and nutritional interventions on diarrhoea and child growth in rural Bangladesh: a cluster randomised controlled trial. Lancet Glob Health 2018;6:e302–15. 29396217 10.1016/S2214-109X(17)30490-4 PMC5809718

[14] Ram PK. Practical Guidance for Measuring Handwashing Behavior: 2013 Update. Water and Sanitation Program, 2013 https://www.wsp.org/sites/wsp.org/files/publications/WSP-Practical-Guidance-Measuring-Handwashing-Behavior-2013-Update.pdf (19 November 2018, date last accessed).

[15] Luby SP, Halder AK, Huda TM, Unicomb L, Johnston RB. Using child health outcomes to identify effective measures of handwashing. Am J Trop Med Hyg 2011;85:882–92. 22049043 10.4269/ajtmh.2011.11-0142 PMC3205635

[16] Ram PK, Halder AK, Granger SP et al Is structured observation a valid technique to measure handwashing behavior? Use of acceleration sensors embedded in soap to assess reactivity to structured observation. Am J Trop Med Hyg 2010;83:1070–76. 21036840 10.4269/ajtmh.2010.09-0763 PMC2963972

[17] Stevens GA, Alkema L, Black RE et al Guidelines for accurate and transparent health estimates reporting: the GATHER statement. PLoS Med 2016;13:e1002056. 27351744 10.1371/journal.pmed.1002056 PMC4924581

[18] GATHER. Guidelines for Accurate and Transparent Health Estimates Reporting]. Gather Statement. http://gather-statement.org/ (16 April 2018, date last accessed).

[19] WHO, UNICEF. *Hygiene* https://washdata.org/monitoring/hygiene (19 October 2017, date last accessed).

[20] WHO. *WHO Regional Groupings* *World Health Stat 2017 Monit Health SDGs Sustain Dev Goals* 2017 http://apps.who.int/iris/bitstream/10665/255336/1/9789241565486-eng.pdf.

[21] World Bank. *World Bank Country and Lending Groups – World Bank Data Help Desk* 2016 https://datahelpdesk.worldbank.org/knowledgebase/articles/906519-world-bank-country-and-lending-groups (19 October 2016, date last accessed).

[22] StataCorp. *Stata Statistical Software: Release 14* College Station, TX: Statacorp, 2015.

[23] R Core Team. R: A Language and Environment for Statistical Computing. Vienna: R Foundation for Statistical Computing, 2015.

[24] Gelman A, Su Y-S. arm: Data Analysis Using Regression and Multilevel/Hierarchical Models. R Package Version 1.9-3. 2016 https://CRAN.R-project.org/package=arm (19 November 2018, date last accessed).

[25] United Nations Population Division. *World Population Prospects 2017.* 2017. https://esa.un.org/unpd/wpp/ (19 October 2017, date last accessed).

[26] De Onis M, Blössner M, Borghi E, Morris R, Frongillo EA. Methodology for estimating regional and global trends of child malnutrition. Int J Epidemiol 2004;33:1260–70. 15542535 10.1093/ije/dyh202

[27] Arlot S, Celisse A. A survey of cross-validation procedures for model selection. Stat Surv 2010;4:40–79.

[28] Curtis VA, Danquah LO, Aunger RV. Planned, motivated and habitual hygiene behaviour: an eleven country review. Health Educ Res 2009;24:655–73. 19286894 10.1093/her/cyp002 PMC2706491

[29] Biran A, Rabie T, Schmidt W, Juvekar S, Hirve S, Curtis V. Comparing the performance of indicators of hand-washing practices in rural Indian households. Trop Med Int Health 2008;13:278–85. 18304276 10.1111/j.1365-3156.2007.02001.x

[30] Vujcic J, Ram PK. PPPHW Program Baseline Data Analysis: Report; Version 4. Buffalo, NY: University at Buffalo, 2013.

[31] EAWAG. *Handwashing Campaigns in Rural and Urban Zimbabwe* 2017 https://www.ranasmosler.com/single-post/2017/07/06/Evaluation-report (6 October 2017, date last accessed).

[32] Barros AJ, Hirakata VN. Alternatives for logistic regression in cross-sectional studies: an empirical comparison of models that directly estimate the prevalence ratio. BMC Med Res Methodol 2003;3:21. 14567763 10.1186/1471-2288-3-21 PMC521200

[33] Zou G. A modified poisson regression approach to prospective studies with binary data. Am J Epidemiol 2004;159:702–06. 15033648 10.1093/aje/kwh090

[34] Williams R. Using Stata’s Margins Command to Estimate and Interpret Adjusted Predictions and Marginal Effects. University of Notre Dame, 2017. https://www3.nd.edu/∼rwilliam/stats/Margins01.pdf (12 October 2017, date last accessed).

[35] Moher D, Liberati A, Tetzlaff J, Altman DG. Preferred reporting items for systematic reviews and meta-analyses: the PRISMA statement. Ann Intern Med 2009;151:264–69. 19622511 10.7326/0003-4819-151-4-200908180-00135

[36] Contzen N, Meili IH, Mosler H-J. Changing handwashing behaviour in southern Ethiopia: a longitudinal study on infrastructural and commitment interventions. Soc Sci Med 2015;124:103–14. 25461867 10.1016/j.socscimed.2014.11.006

[37] Borja-Vega C, Briceño B, Garcia V. Senegal - WSP Global Scaling up Handwashing Behavior Impact Evaluation 2009-2011, Baseline and Endline Surveys. World Bank, 2016 http://microdata.worldbank.org/index.php/catalog/2557 (19 November 2018, date last accessed).

[38] Briceño B, Coville A, Martinez S. Promoting Handwashing and Sanitation: Evidence From a Large-scale Randomized Trial in Rural Tanzania. Washington, DC: World Bank, 2015.

[39] Galiani S, Gertler P, Ajzenman N, Orsola-Vidal A. Promoting handwashing behavior: the effects of large-scale community and school-level interventions. Health Econ 2016;25:1545–59. 26461811 10.1002/hec.3273

[40] Qadri F, Ali M, Chowdhury F et al Feasibility and effectiveness of oral cholera vaccine in an urban endemic setting in Bangladesh: a cluster randomised open-label trial. Lancet 2015;386:1362–71. 26164097 10.1016/S0140-6736(15)61140-0

[41] Chase C, Do Q-T. Handwashing Behavior Change at Scale: Evidence From a Randomized Evaluation in Vietnam. Washington, DC: World Bank, 2012.

[42] Higgins J, Thompson SG, Deeks JJ, Altman DG. Measuring inconsistency in meta-analyses. BMJ 2003;327:557–60. 12958120 10.1136/bmj.327.7414.557 PMC192859

[43] Viechtbauer W, Cheung MW-L. Outlier and influence diagnostics for meta-analysis. Res Synth Methods 2010;1:112–25. 26061377 10.1002/jrsm.11

[44] Jeong J, Choi J, Jeong I, Paek K, In H, Park K. [A nationwide survey on the hand washing behavior and awareness]. J Prev Med Public Health 2007;40:197–204. 17577074 10.3961/jpmph.2007.40.3.197

[45] WHO, UNICEF. Progress on Drinking Water, Sanitation and Hygiene: 2017 Update and SDG Baselines. Geneva: WHO, UNICEF, 2017.

[46] Curtis V, Biran A, Deverell K, Hughes C, Bellamy K, Drasar B. Hygiene in the home: relating bugs and behaviour. Soc Sci Med 2003;57:657–72. 12821014 10.1016/s0277-9536(02)00409-4

[47] Curtis V. Hand Washing at LSHTM—The Shocking Truth. Seattle, WA: Institute for Health Metrics and Evaluation, 2004.

[48] Wolf J, Hunter PR, Freeman MC et al Impact of drinking water, sanitation and hand washing with soap on childhood diarrhoeal disease: updated meta-analysis and –regression. Trop Med Int Health. 2018 https://onlinelibrary.wiley.com/doi/abs/10.1111/tmi.13051 (18 March 2018, date last accessed). 10.1111/tmi.13051 29537671

[49] Aiello AE, Coulborn RM, Perez V, Larson EL. Effect of hand hygiene on infectious disease risk in the community setting: a meta-analysis. Am J Public Health 2008;98:1372–81. 18556606 10.2105/AJPH.2007.124610 PMC2446461

