# Supplementary material for: Handwashing with soap after potential faecal contact: global, regional and country estimates
Source: Int J Epidemiol. 2018 Dec 10;48(4):1204–18. doi: 10.1093/ije/dyy253 (PMC6693803; doi:10.1093/ije/dyy253)
Supplement: dyy253_Supplementary_Data [file dyy253_supplementary_data.zip › dyy253-Suppl_data/Supplementary_File.docx]

Appendix

The appendix is divided by objective for greater clarity which corresponds to the objectives of the manuscript.

Table of contents

[A.0.1: Methods used by objective (Table) 3](#_Toc530470315)

[A.0.2: GATHER checklist (separate file) 3](#_Toc530470316)

[Appendix Objective 1 3](#_Toc530470317)

[A.1.1 Regional groupings 3](#_Toc530470318)

[A.1.2 Modelling of designated handwashing facility presence 5](#_Toc530470319)

[Appendix Objective 2 5](#_Toc530470320)

[A.2.1 Study characteristics, prevalence estimates and citations for studies in high-income countries included in meta-analysis (Table) 6](#_Toc530470321)

[A.2.2 Forest plot of HWWS observation studies by country income status (Figure) 8](#_Toc530470322)

[A.2.3: Modelling the association between presence of a designated handwashing facility and observed HWWS 9](#_Toc530470323)

[A.2.4 Studies excluded from the analysis of the association between presence of a designated handwashing facility and HWWS (Table) 9](#_Toc530470324)

[Appendix Objective 3 10](#_Toc530470325)

[A.3.1: Adaptation of confidence intervals when projecting from regional to country level ($\boldsymbol{p}\text{HWWS}\text{, HW place },\boldsymbol{p}\text{HWWS}\text{, no HW place })$ 10](#_Toc530470326)

[A.3.2: Confidence interval estimation for country, regional and global estimates of HWWS ($\boldsymbol{p}\text{HWWS})$ 10](#_Toc530470327)

[A.3.3: Partial derivatives of Equation (1) used in Equation (A5) for the different proportions $\boldsymbol{pi}$ (Table) 12](#_Toc530470328)

[A.3.4: Country estimates for HWWS after potential faecal contact for the year 2015 (Excel file) 12](#_Toc530470329)

[References 13](#_Toc530470330)

# A.0.1: Methods used by objective (Table)

| **Objective** | **Input data** | **Method used** | **Output** |
| --- | --- | --- | --- |
| Objective 1: Quantifying the presence of a designated handwashing facility | 115 country-representative household survey data points from 77 countries on percentages of households that have a handwashing facility with soap and water available on premises | Multi-level modelling (two- level linear model) | Modelled estimates of percentage of population having a designated handwashing facility for 194 countries and one territory |
| Objective 2: Assessing the association between presence of a designated handwashing facility and observed HWWS | not applicable | Systematic review | Studies on observed handwashing frequency (structured observations) after potential faecal contact |
|  | LMIC: 9 datasets from 8 countries with household-level data on both HWWS practices and presence of a designated handwashing facility | Multi-level modelling (three- level Poisson model) | Association between presence of a designated handwashing facility and observed HWWS, HWWS prevalence by having a designated handwashing facility and region |
|  | HIC:  15 studies from 6 countries | Meta-analysis | Pooled HWWS prevalence |
| Objective 3: Deriving country, regional and global estimates of HWWS prevalence after potential faecal contact | LMIC: Output objective 1; output objective 2 (LMIC) | Calculation using modelled presence of a designated handwashing facility (objective 1) and its association between observed HWWS (objective 2). | HWWS prevalence estimates after potential faecal contact by country |
|  | HIC: Output objective 2 (HIC) | not applicable |  |

HIC: high-income countries, LMIC: low- and middle-income countries

# A.0.2: GATHER checklist (separate file)

# Appendix Objective 1

## A.1.1 Regional groupings

**WHO African Region (without high-income countries)**: Algeria, Angola, Benin, Botswana, Burkina Faso, Burundi, Cabo Verde, Cameroon, Central African Republic, Chad, Comoros, Congo, Côte d’Ivoire, Democratic Republic of the Congo, Equatorial Guinea, Eritrea, Ethiopia, Gabon, Gambia, Ghana, Guinea, Guinea-Bissau, Kenya, Lesotho, Liberia, Madagascar, Malawi, Mali, Mauritania, Mauritius, Mozambique, Namibia, Niger, Nigeria, Rwanda, Sao Tome and Principe, Senegal, Sierra Leone, South Africa, South Sudan, Swaziland, Togo, Uganda, United Republic of Tanzania, Zambia, Zimbabwe.

**WHO Region of the Americas (without high-income countries)**: Argentina, Belize, Bolivia (Plurinational State of), Brazil, Colombia, Costa Rica, Cuba, Dominica, Dominican Republic, Ecuador, El Salvador, Grenada, Guatemala, Guyana, Haiti, Honduras, Jamaica, Mexico, Nicaragua, Panama, Paraguay, Peru, Saint Lucia, Saint Vincent and the Grenadines, Suriname, Venezuela (Bolivarian Republic of).

**WHO South-East Asia Region (without high-income countries)**: Bangladesh, Bhutan, Democratic People’s Republic of Korea, India, Indonesia, Maldives, Myanmar, Nepal, Sri Lanka, Thailand, Timor-Leste.

**WHO European Region (without high-income countries)**: Albania, Armenia, Azerbaijan, Belarus, Bosnia and Herzegovina, Bulgaria, Georgia, Kazakhstan, Kyrgyzstan, Montenegro, Republic of Moldova, Romania, Russian Federation, Serbia, Tajikistan, The former Yugoslav Republic of Macedonia, Turkey, Turkmenistan, Ukraine, Uzbekistan.

**WHO Eastern Mediterranean Region (without high-income countries)**: Afghanistan, Djibouti, Egypt, Iran (Islamic Republic of), Iraq, Jordan, Lebanon, Libya, Morocco, Pakistan, Somalia, Sudan, Syrian Arab Republic, Tunisia, Yemen.

**WHO Western Pacific Region (without high-income countries)**: Cambodia, China, Cook Islands, Fiji, Kiribati, Lao People’s Democratic Republic, Malaysia, Marshall Islands, Micronesia (Federated States of), Mongolia, Niue, Palau, Papua New Guinea, Philippines, Samoa, Solomon Islands, Tonga, Tuvalu, Vanuatu, Viet Nam. (1)

**High-income countries**: Andorra, Antigua and Barbuda, Australia, Austria, Bahamas, Bahrain, Barbados, Belgium, Brunei Darussalam, Canada, Chile, Croatia, Cyprus, Czech Republic, Denmark, Estonia, Finland, France, Germany, Greece, Hungary, Iceland, Ireland, Israel, Italy, Japan, Kuwait, Latvia, Lithuania, Luxembourg, Malta, Monaco, Nauru, Netherlands, New Zealand, Norway, Oman, Poland, Portugal, Qatar, Republic of Korea, Saint Kitts and Nevis, San Marino, Saudi Arabia, Seychelles, Singapore, Slovakia, Slovenia, Spain, Sweden, Switzerland, Trinidad and Tobago, United Arab Emirates, the United Kingdom, the United States of America, Uruguay. (2)

## A.1.2 Modelling of designated handwashing facility presence

The two-level linear model is shown in Equation (A1),

|  | $\text{logit}\left( Y_{ij} \right)=\beta_{0}+\beta_{1}X_{1ij}+\sum_{k=2}^{6} \beta_{k}X_{kj}+\sum_{l=7}^{9} \beta_{l}X_{lj}+\mu_{j}+\varepsilon_{ij}$ | (A1) |
| --- | --- | --- |

where $\text{logit}\left( Y_{ij} \right)$ is the proportion of presence of a designated handwashing facility on the logit scale for year *i* and country *j* , $\beta_{0}$ is the overall mean intercept of $Y$ across groups, $\beta_{1}$ is the regression coefficient for the predictor year $X_{1ij}$ (defined at level 1: individual observations-level), $\beta_{k}$ are the regression coefficients for the regional covariates $X_{kj}$ (defined at level 2: country-level), $\beta_{l}$ are the regression coefficients for the income level covariates $X_{lj}$ (defined at level 2), $\mu_{j}$ are the residuals on the country-level (group-level residuals) and $\varepsilon_{ij}$ are the residuals at the level of the observations (individual observations-level residuals).

For the purpose of this analysis, Algeria was not coded as belonging to the WHO African Region (AFR) but as WHO Eastern Mediterranean Region (EMR) given that the exposure pattern is more similar to EMR that AFR countries.

# Appendix Objective 2

## A.2.1 Study characteristics, prevalence estimates and citations for studies in high-income countries included in meta-analysis (Table)

| **Country** | **Study** | **HWWS occurred** | **potential faecal contacts** | **proportion HWWS** | **year of publication** | **setting** |
| --- | --- | --- | --- | --- | --- | --- |
| Austria | Faiz, K. W., Sundseth, A., & Altmann, M. (2014). Hand hygiene among neurologists attending a congress. Am J Infect Control, 42(2), e27-28. doi:10.1016/j.ajic.2013.10.013 | 148 | 200 | 0.74 | 2014 | public (toilet at conference) |
| Korea (Republic of) | Lee MS, Hong SJ, Kim YT. Handwashing with soap and national handwashing projects in Korea: focus on the National Handwashing Survey, 2006-2014. Epidemiol Health. 2015;37:e2015039. | 330 | 1120 | 0.29 | 2015 | public (public toilets) |
| Korea (Republic of) | Jeong, J. S., Choi, J. K., Jeong, I. S., Paek, K. R., In, H. K., & Park, K. D. (2007). [A nationwide survey on the hand washing behavior and awareness]. J Prev Med Public Health, 40(3), 197-204. | 488 | 2800 | 0.17 | 2007 | public (public toilets) |
| New Zealand | Garbutt, C., Simmons, G., Patrick, D., & Miller, T. (2007). The public hand hygiene practices of New Zealanders: a national survey. N Z Med J, 120(1265), U2810. | 859 | 1200 | 0.72 | 2007 | public (public toilets) |
| Poland | Malinowska-Borowska, J., Wolny, M., & Krause, M. (2015). Hand washing practices of the residents of Silesia (Poland) based on observations in public toilets. Journal of Water Sanitation and Hygiene for Development, 5(1), 107-114. | 204 | 400 | 0.51 | 2015 | public (public toilets) |
| UK | Curtis V, Biran A, Deverell K, Hughes C, Bellamy K, Drasar B. Hygiene in the home: relating bugs and behaviour. Soc Sci Med, 57(4), 657-672 | 31 | 54 | 0.57 | 2003 | household-level |
| UK | Judah G, Aunger R, Schmidt W, Michie S, Granger S, Curtis V. Experimental Pretesting of Hand-Washing Interventions in a Natural Setting. American Journal of Public Health. 2009;99(S2):S405-S411. | 92826 | 198000 | 0.47 | 2009 | public (public toilets) |
| UK | Curtis V. Hand washing at LSHTM - the shocking truth. 2004 | 146 | 277 | 0.53 | 2004 | public (toilet in university) |
| USA | Allwood P. Handwashing among public restroom users at the Minnesota State Fair. Minnesota Department of Health; 2004. | 611 | 1175 | 0.52 | 2004 | public (public toilets) |
| USA | Borchgrevink, C. P., Cha, J., & Kim, S. (2013). Hand washing practices in a college town environment. J Environ Health, 75(8), 18-24. | 2509 | 3749 | 0.67 | 2003 | public (public toilets) |
| USA | Nichols AL. Actual vs. reported behavior - Increasing handwashing in public restrooms. Swiss Journal of Psychology. 2014;73(1):41-6. | 49 | 111 | 0.44 | 2014 | public (toilet in university) |
| USA | Mackert, M., Liang, M. C., & Champlin, S. (2013). "Think the sink:" Preliminary evaluation of a handwashing promotion campaign. Am J Infect Control, 41(3), 275-277. | 583 | 1005 | 0.58 | 2013 | public (toilet in university) |
| USA | Anderson, J. L., Warren, C. A., Perez, E., Louis, R. I., Phillips, S., Wheeler, J., . . . Misra, R. (2008). Gender and ethnic differences in hand hygiene practices among college students. Am J Infect Control, 36(5), 361-368. | 699 | 1400 | 0.50 | 2008 | public (toilet in university) |
| USA | Drankiewicz, D., & Dundes, L. (2003). Handwashing among female college students. Am J Infect Control, 31(2), 67-71. | 38 | 100 | 0.38 | 2003 | public (toilet in university) |
| USA | Johnson, H. D., Sholcosky, D., Gabello, K., Ragni, R., & Ogonosky, N. (2003). Sex differences in public restroom handwashing behavior associated with visual behavior prompts. Percept Mot Skills, 97(3 Pt 1), 805-810. | 42 | 85 | 0.49 | 2013 | public (toilet in university) |

## A.2.2 Forest plot of HWWS observation studies by country income status (Figure)

|  |
| --- |

## A.2.3: Modelling the association between presence of a designated handwashing facility and observed HWWS

The model (not including effect modification by region) is shown in Equation (A2),

|  | $\log\left( \frac{\pi_{ijk}}{1-\pi_{ijk}} \right)=\beta_{0}+\beta_{1}X_{1jk}+\sum_{s=2}^{3} \beta_{s}X_{sk}+\vartheta_{k}+\mu_{jk}$ | (A2) |
| --- | --- | --- |

where $\log\left( \frac{\pi_{ijk}}{1-\pi_{ijk}} \right)$ is the log odds of HWWS of observed potential handwashing occasion $i$, household $j$, and country $k$. $\beta_{0}$ is the overall mean intercept between the log odds and the covariates, $\beta_{1}$ is the coefficient for having a designated handwashing facility $X_{1jk}$ (defined at level 2: household), $\beta_{s}$ are the two regression coefficients for the regional covariates $X_{sk}$ (defined at level 3: country; only three WHO regions are covered by the included datasets), $\vartheta_{k}$ are the residuals at country level and $\mu_{jk}$ the residuals at the level of the household.

## A.2.4 Studies excluded from the analysis of the association between presence of a designated handwashing facility and HWWS (Table)

| **country (WHO region)** | **year** | **setting** | **reasons for exclusion** | **reference** |
| --- | --- | --- | --- | --- |
| Bangladesh (SEAR) | 2007 | rural | no access to data | (3) |
| Bangladesh (SEAR) | 2009/2010 | urban | no access to data | (4) |
| Kyrgyzstan (EUR) | 2000 | rural | 1. presence of soap was not assessed as presence at the handwashing station but meant soap anywhere in/at the house; 2. reported is presence of a washstand (device with a tank and a tap) which is not the same as presence of a designated place for handwashing | (5) |
|  |  |  |  |  |

# Appendix Objective 3

## A.3.1: Adaptation of confidence intervals when projecting from regional to country level ($\boldsymbol{p}_{\text{HWWS}\text{, HW place }}\mathbf{,}\boldsymbol{p}_{\text{HWWS}\text{, no HW place }}\mathbf{)}$

The estimate for $p_{\text{HWWS, HW place}}$ and $p_{\text{HWWS, no HW place}}$ at country level are taken from model results at regional level. When projecting confidence intervals (CIs) from regional to country level an information dilution effect had to be taken into account, i.e. an increase of the CIs. Otherwise a re-aggregation (with its inherent reduction of CIs see Equation (A3) ) would result in lower CIs as initially gained from the model. A correction factor to account for this can be derived from the relation between regional and country specific standard errors:

|  | $\sigma_{reg}=\sqrt{\frac{\sum_{i=1}^{n} N_{i}^{2}\sigma_{i}^{2}}{\left( \sum_{i=1}^{n} N_{i} \right)^{2}}}$ | (A3) |
| --- | --- | --- |

assuming equal country specific standard errors $\sigma_{i}$ in one region:

|  | $\sigma_{reg}=\sigma_{i}\frac{\sqrt{\sum_{i=1}^{n} N_{i}^{2}}}{\sum_{i=1}^{n} N_{i}}$ $\sigma_{i}=\sigma_{reg}\frac{\sum_{i=1}^{n} N_{i}}{\sqrt{\sum_{i=1}^{n} N_{i}^{2}}}.$ | (A4) |
| --- | --- | --- |

## A.3.2: Confidence interval estimation for country, regional and global estimates of HWWS ($\boldsymbol{p}_{\text{HWWS}}\mathbf{)}$

**Confidence intervals at country-level**

The confidence intervals (CI) of $p_{\text{HWWS}}$

|  | $CI\left( p_{\text{HWWS}} \right)=\text{logit}^{-1}\left( \text{logit}\left( p_{\text{HWWS}} \right)\pm1.96\cdot\Delta\text{logit}\left( p_{\text{HWWS}} \right) \right)$ | (A5) |
| --- | --- | --- |

are calculated under the assumption of independent normal distributions on the logit scale for all proportions. The logit function is

|  | $\text{logit}\left( p \right)=\ln\left( \frac{p}{1-p} \right).$ | (A6) |
| --- | --- | --- |

The (symmetric) standard error of $p_{\text{HWWS}}$ on the logit scale $\Delta\text{logit}\left( p_{\text{HWWS}} \right)$ is calculated using the error propagation method for the individual contributions of the different proportions $p_{i}$ in Equation (1) (i.e., $p_{\text{HWWS, HW place}}$, $p_{\text{HWWS, no HW place}}$ and $p_{\text{HW place}}$):

|  | $\Delta\text{logit}\left( p_{\text{HWWS}} \right)\approx\sqrt{\sum_{i} \left( \frac{\partial\text{logit}\left( p_{\text{HWWS}} \right)}{\partial\text{logit}\left( p_{i} \right)}\Delta\text{logit}\left( p_{i} \right) \right)^{2}}\approx\sqrt{\sum_{i} \left( \frac{\partial\text{logit}\left( p_{\text{HWWS}} \right)}{\partial p_{\text{HWWS}}}\frac{\partial p_{\text{HWWS}}}{\partial p_{i}}\frac{\partial p_{i}}{\partial\text{logit}\left( p_{i} \right)} \Delta\text{logit}\left( p_{i} \right) \right)^{2}}$ $=\sqrt{\sum_{i} \left( \frac{\partial p_{\text{HWWS}}}{\partial p_{i}}\frac{\partial\text{logit}\left( p_{\text{HWWS}} \right)}{\partial p_{\text{HWWS}}}\left( \frac{\partial\text{logit}\left( p_{i} \right)}{\partial p_{i}} \right)^{-1}\Delta\text{logit}\left( p_{i} \right) \right)^{2}}.$ | (A7) |
| --- | --- | --- |

The derivative of the logit function

|  | $\frac{\text{d}}{\text{d}p}\text{logit}\left( p \right)=\frac{1}{p\left( 1-p \right)}$ | (A8) |
| --- | --- | --- |

yields

|  | $\Delta\text{logit}\left( p_{\text{HWWS}} \right)\approx\sqrt{\sum_{i} \left( \frac{\partial p_{\text{HWWS}}}{\partial p_{i}}\frac{p_{i}\left( 1-p_{i} \right)}{p_{\text{HWWS}}\left( 1-p_{\text{HWWS}} \right)} \Delta\text{logit}\left( p_{i} \right) \right)^{2}}.$ | (A9) |
| --- | --- | --- |

The individual standard errors on the logit scale of the different proportions $\Delta\text{logit}\left( p_{i} \right)$ are calculated with the inverse approach of Equation (A3):

|  | $\Delta\text{logit}\left( p_{i} \right)=\frac{1}{2\cdot1.96}\left[ \text{logit}\left( UCL\left( p_{i} \right) \right)-\text{logit}\left( LCL\left( p_{i} \right) \right) \right].$ | (A10) |
| --- | --- | --- |

The partial derivatives of $p_{\text{HWWS}}$ for the different proportions are given in Table A.4.2.

**Confidence intervals at regional and global level**

Confidence intervals at regional and global level were calculated from confidence intervals at country-level using standard formulae. The standard error at regional and global level was estimated with an approach using the delta method which is described in detail by De Onis et al. (6) for a similar context and in Equation (A3). As we do not know the total number of potential faecal contacts by country and region we assume an equal average number of faecal contacts by person across countries and regions and use total population by country and region to calculate the regional and global confidence intervals.

## A.3.3: Partial derivatives of Equation (1) used in Equation (A5) for the different proportions $\boldsymbol{p}_{\boldsymbol{i}}$ (Table)

| $p_{i}$ | $\frac{\partial p_{\text{HWWS}}}{\partial p_{i}}$ |
| --- | --- |
| $p_{\text{HWWS, HW place}}$ | $p_{\text{HW place}}$ |
| $p_{\text{HWWS, no HW place}}$ | $1-p_{\text{HW place}}$ |
| $p_{\text{HW place}}$ | $p_{\text{HWWS, HW place}}-p_{\text{HWWS, no HW place}}$ |

## A.3.4: Country estimates for HWWS after potential faecal contact for the year 2015 (Excel file)

see separate Excel file

# References

1. WHO. WHO regional groupings. In: World Health Statistics 2017: Monitoring Health for the SDGs, Sustainable Development Goals [Internet]. 2017. p. 103. Available from: http://apps.who.int/iris/bitstream/10665/255336/1/9789241565486-eng.pdf

2. World Bank. World Bank Country and Lending Groups – World Bank Data Help Desk [Internet]. 2017 [cited 2017 Nov 15]. Available from: https://datahelpdesk.worldbank.org/knowledgebase/articles/906519

3. Halder AK, Tronchet C, Akhter S, Bhuiya A, Johnston R, Luby SP. Observed hand cleanliness and other measures of handwashing behavior in rural Bangladesh. BMC Public Health. 2010;10(1):1.

4. Doshi S, Silk BJ, Dutt D, Ahmed M, Cohen AL, Taylor TH, et al. Household-level risk factors for influenza among young children in Dhaka, Bangladesh: a case–control study. Trop Med Int Health. 2015 Jun 1;20(6):719–29.

5. Biran A, Tabyshalieva A, Salmorbekova Z. Formative research for hygiene promotion in Kyrgyzstan. Health Policy Plan. 2005;20(4):213–221.

6. De Onis M, Blössner M, Borghi E, Morris R, Frongillo EA. Methodology for Estimating Regional and Global Trends of Child Malnutrition. Int J Epidemiol. 2004 Jan 12;33(6):1260–70.
