# Supplementary material for: Handwashing with soap after potential faecal contact: global, regional and country estimates
Source: Int J Epidemiol. 2018 Dec 10;48(4):1204–18. doi: 10.1093/ije/dyy253 (PMC6693803; doi:10.1093/ije/dyy253)
Supplement: dyy253_Supplementary_Data [file dyy253_supplementary_data.zip › dyy253-Suppl_data/Supplementary_Data.docx]

**
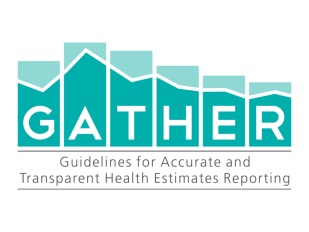
Checklist of information that should be included in new reports of global health estimates**

| Item # | Checklist item | Reported in the following section |
| --- | --- | --- |
| Objectives and funding | | |
| 1 | Define the indicator(s), populations (including age, sex, and geographic entities), and time period(s) for which estimates were made. | indicator 1: designated handwashing facility 🡪 methods: objective 1: 1^st^ and 2^nd^ paragraph, and  🡪 methods: objective 2: multi-level analysis…: exposure definition  indicator 2: HWWS after potential faecal contact 🡪 methods: objective 2: Multi-level analysis: outcome definition |
| 2 | List the funding sources for the work. | 🡪 Acknowledgements |
| Data Inputs | | |
| *For all data inputs from multiple sources that are synthesized as part of the study:* | | |
| 3 | Describe how the data were identified and how the data were accessed. | indicator 1: designated handwashing facility 🡪 methods: objective 1: 1^st^ paragraph  indicator 2: HWWS after potential faecal contact 🡪 methods: objective 2: systematic review… |
| 4 | Specify the inclusion and exclusion criteria. Identify all ad-hoc exclusions. | indicator 1: designated handwashing facility: NA (the WHO/UNICEF (JMP) database was used without any restrictions  indicator 2: HWWS after potential faecal contact 🡪 methods: objective 2: systematic review…: 2^nd^ paragraph, and  🡪 methods: objective 2: multi-level analysis…: 1^st^ paragraph, Appendix A.2.4 |
| 5 | Provide information on all included data sources and their main characteristics. For each data source used, report reference information or contact name/institution, population represented, data collection method, year(s) of data collection, sex and age range, diagnostic criteria or measurement method, and sample size, as relevant. | indicator 1: designated handwashing facility 🡪 methods: objective 1: 1^st^ paragraph, and  🡪Appendix A.3.4  indicator 2: HWWS after potential faecal contact 🡪 Figure 2, Table 3, Appendix A.2.1, A.2.2 and A.3.4 |
| 6 | Identify and describe any categories of input data that have potentially important biases (e.g., based on characteristics listed in item 5). | indicator 1: designated handwashing facility: NA (input data are from standardized, country-representative observations)  indicator 2: HWWS after potential faecal contact 🡪 discussion: strengths and limitations: objective 2 |
| *For data inputs that contribute to the analysis but were not synthesized as part of the study:* | | |
| 7 | Describe and give sources for any other data inputs. | indicator 1: designated handwashing facility 🡪 methods: objective 1: 2^nd^ and 4^th^ paragraph, and  🡪 Appendix A.1.1  indicator 2: HWWS after potential faecal contact: NA |
| *For all data inputs:* | | |
| 8 | Provide all data inputs in a file format from which data can be efficiently extracted (e.g., a spreadsheet rather than a PDF), including all relevant meta-data listed in item 5. For any data inputs that cannot be shared because of ethical or legal reasons, such as third-party ownership, provide a contact name or the name of the institution that retains the right to the data. | indicator 1: designated handwashing facility: the JMP global database is used, this data can be downloaded from <https://washdata.org/>  indicator 2: HWWS after potential faecal contact: Table 3 (low- and middle-income countries), Tables A.2.1 (high-income countries) |
| Data analysis | | |
| 9 | Provide a conceptual overview of the data analysis method. A diagram may be helpful. | Appendix A.0.1 (overview of all analysis methods) |
| 10 | Provide a detailed description of all steps of the analysis, including mathematical formulae. This description should cover, as relevant, data cleaning, data pre-processing, data adjustments and weighting of data sources, and mathematical or statistical model(s). | indicator 1: designated handwashing facility 🡪 methods: objective 1: 2^nd,^ 3^rd^ and 4^th^ paragraph, and  🡪 Appendix A.1.2  indicator 2: HWWS after potential faecal contact 🡪methods: objective 2: multi-level analysis: statistical analysis, and  🡪 objective 3: 1^st^ paragraph, and  🡪 Appendix A.2.3, A.3.1, A.3.2, A.3.3 |
| 11 | Describe how candidate models were evaluated and how the final model(s) were selected. | indicator 1: designated handwashing facility 🡪the model is an adaptation from previously developed models for different WaSH components which have been thoroughly tested and evaluated (e.g., https://www.ncbi.nlm.nih.gov/pubmed/23428550) |
| 12 | Provide the results of an evaluation of model performance, if done, as well as the results of any relevant sensitivity analysis. | indicator 1: designated handwashing facility 🡪 results: objective 1: modelled results …: 2^nd^ and 3^rd^ paragraph  indicator 2: HWWS after potential faecal contact 🡪 results: objective 2: multi-level analysis: 5^th^ paragraph |
| 13 | Describe methods for calculating uncertainty of the estimates. State which sources of uncertainty were, and were not, accounted for in the uncertainty analysis. | indicator 1: designated handwashing facility 🡪 methods: objective 1: 3^rd^ and 4^th^ paragraph, and  🡪Appendix A.3.1  indicator 2: HWWS after potential faecal contact 🡪 methods: objective 3 |
| 14 | State how analytic or statistical source code used to generate estimates can be accessed. | 🡪 methods: 1^st^ paragraph |
| Results and Discussion | | |
| 15 | Provide published estimates in a file format from which data can be efficiently extracted. | Table 2, Appendix A.3.4 |
| 16 | Report a quantitative measure of the uncertainty of the estimates (e.g. uncertainty intervals). | indicator 1: designated handwashing facility 🡪 Table 2  indicator 2: HWWS after potential faecal contact 🡪 Tables 4 and 5 |
| 17 | Interpret results in light of existing evidence. If updating a previous set of estimates, describe the reasons for changes in estimates. | 🡪Discussion: 3^rd^ paragraph |
| 18 | Discuss limitations of the estimates. Include a discussion of any modelling assumptions or data limitations that affect interpretation of the estimates. | 🡪 Discussion: Strengths and limitations (objectives 1-3) |

*This checklist should be used in conjunction with the GATHER statement and Explanation and Elaboration document, found on gather-statement.org*
